# The impact of management practices and industry 4.0 technologies on supply chain sustainability: A systematic review

**DOI:** 10.1016/j.heliyon.2024.e36421

**Published:** 2024-08-22

**Authors:** Manaf Al-Okaily, Hassan Younis, Aws Al-Okaily

**Affiliations:** a School of Business, Jadara University, Irbid, Jordan; b Business School, German Jordanian University, Amman, Jordan

**Keywords:** Digital technologies, Management practices, Industry 4.0 technologies, A systematic review, Supply chain management, Sustainability

## Abstract

This systematic review addresses a significant gap in the existing literature by examining the intricate relationship between management practices and Industry 4.0 technologies in shaping supply chain sustainability. While prior studies have explored their individual impacts, this review synthesizes and categorizes findings to identify nuanced trends that contribute to supply chain efficiency, waste reduction, and environmental footprints. To achieve the goal of the study, a rigorous search strategy was employed to select peer-reviewed journal articles focusing on total quality management, just-in-time, vendor-managed inventory, lean, manufacturer-led decentralized systems, blockchain, the internet of things, and big data in the context of supply chain sustainability. The selected studies underwent a thorough evaluation to ensure quality and relevance. The findings highlight key insights: the adoption of management practices, particularly total quality management and just-in-time, significantly contributes to reducing waste, enhancing efficiency, and minimizing environmental footprints across supply chains. Simultaneously, the integration of industry 4.0 technologies like blockchain, the Internet of things, and big data empowers data-driven decision-making, transparency, and traceability, amplifying sustainability efforts. In conclusion, this review contributes a novel perspective by synthesizing, categorizing, and analyzing the impact of management practices and Industry 4.0 technologies on supply chain sustainability. Its findings offer valuable insights for addressing contemporary challenges and advancing sustainable practices amid dynamic global scenarios.

## 1 Introduction

In the face of escalating pressure on organizations to optimize natural resource consumption, minimize waste generation, and mitigate carbon footprints, businesses are diligently exploring avenues for sustained competitiveness [1]. This imperative compels organizations to align their sustainability initiatives with their supply chain partners, fostering shared understanding and collaborative achievement of desired outcomes [2]. Furthermore, contemporary supply chains bear considerable responsibility for climate change-related impacts stemming from diverse processes, spanning from raw material extraction through goods transportation to end-of-life product disposal. Life cycle assessment (LCA) emerged from pivotal studies in the late 1960s and early 1970s, marking the initial strides toward understanding the comprehensive environmental implications of products. Similarly, the "Cradle to Cradle" concept, as introduced by McDonough and Braungart [3] emphasized the integration of reusability and responsible disposal measures during the manufacturing stage, aligning with the principles of sustainability and contributing to the United Nations' Sustainable Development Goals [4]. These frameworks play a pivotal role in guiding supply chain practices towards achieving not only environmental sustainability but also addressing broader social and economic challenges outlined in the Sustainable Development Goals (SDGs).

In response, businesses face scrutiny from an array of stakeholders, ranging from governmental bodies and non-governmental organizations (NGOs) to shareholders and customers, each increasingly demanding sustainable products and services [5]. On the other hand, enterprises are driven to adopt sustainable practices that have a positive green multiplier effect [6], leading to triple-bottom-line advancement in line with regulatory mandates, environmental neutrality promotion [7], and improved social and commercial performance. Nevertheless, alongside the enablers and incentives for sustainable practice adoption, a host of challenges and barriers persist that could impede effective implementation. This complexity warrants in-depth exploration, positioning the field for further inquiry.

In this context, this study aims to conduct a comprehensive review of pertinent publications addressing sustainability and supply chains, with a focus on how specific management practices as well as new technologies impact the sustainable performance of any organization. Our objectives encompass the identification of recurring themes to guide future research, the elucidation of pivotal factors influencing businesses' adoption decisions, and the proposal of insights derived from key theories to facilitate the integration of sustainability practices.

In the following sections, this paper will be structured as follows: Section 2 delves into the existing literature at the intersection of sustainability and supply chains. Section 3 outlines the adopted methodology for this review. Section 4 presents the research findings, and Section 5 concludes by summarizing the results, addressing limitations, and delineating potential future research directions.

## 2 Literature review

Sustainability has become a huge buzzword, popping up in many scholarly and practical works across different domains and gaining high attention as a result of raising public awareness about climate change. Although many human-related activities contribute to climate change, supply chains play a significant role in many sustainability-associated concerns. For example, according to the International Energy Agency's report on global CO2 emissions (2019), goods transportation in 2019 accounted for 24 % of total CO2 emissions. Furthermore, 50 % of all CO2 emissions worldwide were caused by the extraction and processing of resources, according to the Global Resources Outlook report, United Nations Environment Programme.

The following sections elaborate on the most commonly used definitions of sustainability and supply chain.

### 2.1 Definition of sustainability

Since the launch of the term “sustainability” by the World Commission on Environment and Development in 1987, there have been many attempts to define sustainability [8]. Nevertheless, the Environmental Protection Agency's (EPA) definition remained the most widely used in this regard, in which sustainability is defined as "development that meets the needs of the present without compromising the ability of future generations to meet their own needs [8]. A few years later, Elkington [9] popularized the phrase "triple bottom line," claiming that sustainability is nothing more than the union of social, environmental, and economic performance. Interestingly, Younis et al. [10] studied the impact of sustainable practices on corporate performance, and they concluded that the operational dimension needs to be considered in any sustainable-related initiative within the supply chain. Therefore, they claimed that sustainability is the implementation of green practices aimed at improving the environmental, social, economic, and operational performance of the supply chain [11]. Table 1 below summarizes several frequently used sustainability definitions.

**Table 1:** Sustainability definitions.

**Definition**	**Source**
"Development that meets the needs of the present without compromising the ability of future generations to meet their own needs."	Brundtland et al. [8].
Sustainability encompasses environmental and social activities that must not harm the economic performance of the firm.	Elkington [9]
Sustainability is the intersection of the environmental, social, and economic performance of a firm.	Elkington [9]
A strategic, transparent integration and achievement of the organization's social, environmental, and economic goals in coordination with other supply chain members	Carter and Easton [11]
Sustainability is the implementation of green practices aimed at improving the environmental, social, economic, and operational performance of the supply chain.	Younis et al. [10]
"Ensuring a better and more sustainable future for all by balancing economic growth, social inclusion, and environmental protection."	The United Nations 2030 Agenda for Sustainable Development (Programme, Long 2019)

### 2.2 Definition of supply chain (SC)

Despite more than thirty years of study, the concept of SC remains ambiguous, with numerous authors offering their own takes on the term. For instance, Beamon [12] defined SC as a structured manufacturing process that turns raw materials into finished goods and subsequently distributes them to end customers. Two decades later, Chow and Heaver [13] took a collaborative view of SC, defining it as the network of firms involved in delivering products to customers, including producers, shippers, retailers, brokers, and transportation service providers. Sometime later, scholars began to look at the supply chain from the standpoint of three flows: data, goods, and money. Evidently, SC was described by Mentzer et al. [14] and Shapiro [15] as the administration of product, cash, and data flows upstream and downstream for the purpose of satisfying consumer requests.

Alternately, other academics have characterized SC from the perspective of both a process and a flow. For instance, Pienaar [16] provided an illustration by stating that SC is the sum of all of the processes that are required to change the raw materials into the completed product and convey the product to the customer. Additionally, some scholars looked at SC through the lens of efficacy and effectiveness, claiming that SC must provide the right products to the right customers in the right quantities, at the right time, in the right place, at the right price, and with the right level of quality [17]. More recently, a sort of agreed definition has been put forward by a group of researchers and institutions, such as Dutta and Hora [18], the SC Council, and the SC Management Professionals Council, which took a more collaborative perspective. According to this definition, the supply chain is defined as a collection of processes and organizations (suppliers, customers, manufacturing sites, distributors, and retailers) engaged in completing customer orders, with the major SC operations being plan, source, make, deliver, and return [[19], [20], [21], [22], [23], [24]]. Table 2 shows a number of the most often used definitions.

**Table 2:** Supply chain definitions.

No.	Definition	Source
1	Raw materials are transformed into finished goods and delivered to end customers through a structured manufacturing process known as a supply chain.	Beamon [12]
2	The group of manufacturers, suppliers, distributors, retailers, and transportation, information, and other logistics management service providers that are engaged in providing goods to consumers.	Chow and Heaver [13]
3	Three or more firms are linked directly to the upstream and downstream flows of products, services, finances, and information. Through these linkages, individual firms gain access to resources, develop capabilities, and impact performance.	Mentzer et al. [14]
4	Dispersed facilities where raw materials, intermediate products, or finished products are acquired, transformed, stored, or sold, and transportation links that connect facilities along which products flow.	Shapiro [15]
5	A general description of the process integration involving organizations to transform raw materials into finished goods and transport them to the end-user.	Pienaar [16]
6	Collaborative inter- and intra-organizational management on the strategic, tactical, and operational business processes to achieve effective and efficient flows of products, information, and funds to provide the maximum value to the end customer at the lowest cost and with the greatest speed	Zhao and Andreas [19]
7	Receiving the appropriate products in the appropriate quantities, at the right time, in the right location, for the proper cost, and in satisfactory condition for the correct customer.	Wu et al. [17]
8	The supply chain not only connects suppliers, manufacturers, and customers; it also connects several layers of suppliers on the upstream side, and similarly, it connects to the ultimate end users who benefit from the value of the product or service on the downstream side.	Dutta and Hora [18]
9	A set of processes and entities (suppliers, customers, factories, distributors, and retailers) that are interested in fulfilling customer orders Plan, source, make, deliver, return, and enable are the main processes of the supply chain.	The Supply Chain Council

### 2.3 Sustainability and supply chain

There is a tremendous amount of scholarly work on the relationship between sustainability and many aspects of the supply chain. For instance, some studies investigated how logistics organizations try to face the recent ecological challenges and the role that emergent green technologies play in making them finally “green”, competitive, and sustainable. Another study examined how social sustainability is considered in the study of supply chain management, thereby identifying key areas for future researchers to develop. Furthermore, Genc and De Giovanni [25] studied the impact of some innovation-led lean programs in a Closed-loop Supply Chain (CLSC) setting. The researchers found that the implementation of a strategic innovation-led lean program entails a compensation effect between the price and the sales that the manufacturer can incline in favor of sustainability.

In the same vein, Modak et al. [4] reviewed existing literature on sustainability practices adoption in supply chain relationships, analyzing studies, and identifying enablers, barriers, and strategies. The authors underscored the significance of sustainability within supply chain dynamics, with a specific emphasis on the buyer-supplier relationship. They emphasized that supplier capability and capacity play pivotal roles in advancing sustainability goals. Moreover, the authors introduced a conceptual framework aimed at fostering sustainable supply chain development. This model delineates key indicators, enablers, and barriers, thereby stressing the imperative for implementing effective relationship management strategies. More recent studies reviewed the mutual relationship between sustainable supply chain (SSC) and supply chain complexity (SCC) drivers. Using delphi and interpretive structural modeling, the authors concluded that market uncertainty, institutional regulations, strategic supplier collaboration, customer pressure, and new technologies are the five key drivers influencing decision-making in the context of SSC and SCC.

A similar study investigated the potential synergy between efficiency-oriented Green Supply Chain Management (GSCM) and technology-driven Green Information Systems (GIS) on corporate sustainability [26]. The researchers found informal alignment between GSCM and GIS improves organizational performance in economic, operational, environmental, and social aspects. They also found that close integration of efficiency-oriented GSCM and technology-driven GIS leads to greater sustainability outcomes. Employee participation in both activities is crucial for informal alignment and green innovation effectiveness. Besides, other studies sought to evaluate sustainability issues in supply chains by reviewing current methods, proposing three approaches, and evaluating sustainability performance considering economic, environmental, and socio-political risks. The researchers introduced an Assessment of Supply Chain Sustainability (ASSC) framework which is a three-step method for collecting and processing sustainability information from suppliers, reducing duplication of effort, accommodating different detail levels, and facilitating social sustainability assessment. Many other factors are also playing a key role in making supply chains more sustainable including stakeholders.

### 2.4.4 Discussion of management practices and industry 4.0 technologies included in the study

Several researchers explored the impact of management practices and new technologies on supply chain sustainability. For example, scholars such as Ruiz-Benitez et al. [27] and Genc and De Giovanni [25] explored the relationship between sustainability and lean practices. Also, additional empirically investigated whether supply chain agility and lean management practices are antecedents of supply chain social sustainability. The researcher found that agility and lean practices are significant antecedents of social sustainability orientation as well as social sustainability performance.

Similarly, Green et al. [28] measured the impact of adopting Just-In-Time (JIT) and Total Quality Management (TQM) practices on the sustainability performance of the supply chain, concluding that there is a positive relationship between both JIT and TQM and sustainability which was mirrored earlier by Younis [26]. More recently, a fertile area of research has been launched to investigate the impact of Industry 4.0 enablers, including artificial intelligence (AI), machine learning (ML), big data (BD), and the Internet of Things (IoT), on supply chain sustainability [[29], [30], [31], [32], [33], [34]]. Evidently, Park and Li [35] assessed how blockchain technology (BC) can facilitate the adoption of sustainable practices. Moreover, Venkatesh et al. [36] explored how blockchain, the internet of things (IoT), and big data analytics can help monitor supply chain sustainability. In the same vein, Liu et al. [37] proposed a theoretical framework for integrating Industry 4.0 technologies into supply chain and logistics management (SSCM). This framework leverages five growing digital technologies, including cloud services, artificial intelligence (AI), big data analytics (BDA), blockchain technology (BT), and the internet of Things (IoT) [[38], [39], [40], [41], [42], [43]]. Additionally, Patidar et al. [44] investigated the interplay between resilience KPIs and their relation to Industry 4.0 and sustainability. Finally, Weraikat et al. [45] examined the relationship between vendor-managed inventory (VMI) and supply chain sustainability. Table 3 provides a succinct overview of their most widely recognized definitions of the main constructs included in this study.

**Table 3:** Definition of main concepts.

Term	Definition
Total Quality Management (TQM)	A management approach focused on long-term success through customer satisfaction. It involves all members of an organization participating in improving processes, products, services, and the culture in which they work.
Just-In-Time (JIT)	An inventory management strategy that aligns raw-material orders from suppliers directly with production schedules. It aims to reduce inventory costs and increase efficiency by receiving goods only as they are needed in the production process.
Vendor-Managed Inventory (VMI)	A supply chain initiative where the supplier is responsible for maintaining the inventory levels of their products at the buyer's location. The supplier manages the inventory, ensuring that the buyer has the right amount of stock at all times.
Lean Management Practices	A systematic method for waste minimization within a manufacturing system without sacrificing productivity. Lean also takes into account waste created through overburden and unevenness in workloads.
Manufacturer-Led Decentralized Systems	Supply chain systems where the manufacturer takes the lead in coordinating activities and decisions across the supply chain network, allowing for more flexibility and responsiveness to changes in demand or supply conditions.
Blockchain	A distributed ledger technology that ensures transparency, security, and traceability of transactions. In supply chains, blockchain can track the journey of products from origin to consumer, ensuring accountability and reducing fraud.
The Internet of Things (IoT)	The network of physical objects embedded with sensors, software, and other technologies to connect and exchange data with other devices and systems over the internet. IoT enhances supply chain visibility, efficiency, and real-time decision-making.
Big Data	Large and complex data sets that are analyzed computationally to reveal patterns, trends, and associations. In supply chain sustainability, big data helps in predicting demand, optimizing routes, reducing waste, and improving overall efficiency.

### 2.5 Research gap

The prevailing research landscape in the realm of supply chain sustainability underscores a critical research gap that demands closer examination. This void pertains to the comprehensive understanding of how Industry 4.0 technologies, including blockchain (BC), the Internet of Things (IoT), and big data (BD), alongside fundamental management practices such as total quality management, just-in-time, vendor-managed inventory, lean practices, and manufacturer-led decentralized systems, impact the sustainability of supply chains. Our study positions itself at the forefront of addressing this research gap by aiming to unravel the intricate dynamics and synergies that emerge from the convergence of these technologies and management practices within the context of supply chain sustainability.

While existing studies have offered insights into individual components, there exists a noticeable dearth in the literature when it comes to a holistic exploration of the effects of Industry 4.0 technologies such as the IoT, blockchain, and big data, and management practices like TQM, JIT, VMI, lean practices, and manufacturer-led decentralized systems. Consequently, our research seeks to fill this void by delving into the nuanced relationships between these technological advancements and established management principles, offering a more profound understanding of how they contribute to or hinder the sustainability objectives of supply chains.

In essence, our investigation is not merely a compilation of isolated assessments but rather a concerted effort to bridge the identified research gap. By comprehensively examining the interplay between Industry 4.0 technologies and management practices, our study endeavors to provide a robust foundation for strategic decision-making in businesses aiming to enhance the sustainability performance of their supply chains. The imperative lies in elucidating how organizations can strategically integrate their proven management techniques with cutting-edge technologies to navigate the complexities of modern supply chains, ultimately contributing to a more sustainable and resilient global supply chain ecosystem.

### 2.6 Research questions

This study will endeavor to answer the two questions below.

Question 1 How do Industry 4.0 technologies (e.g., blockchain, IoT, big data) and management practices (e.g., TQM, JIT, VMI, lean, MLDS) impact different dimensions of supply chain sustainability (e.g., environmental, economic, social) across diverse industry sectors?

Question 2 In consideration of the above, what challenges, enablers, facilitators, and benefits emerge in the pursuit of sustainability across different performance dimensions in diverse industry sectors?

In addressing the identified research gap, our selected research questions contribute to a more comprehensive understanding of the complex interplay between Industry 4.0 technologies and traditional management practices within the context of supply chain sustainability. The first question delves into the specific impacts on various dimensions of sustainability across diverse industry sectors, bridging the gap in the literature by exploring the nuanced relationships between technology and sustainability outcomes. Meanwhile, the second question offers a practical perspective, elucidating the challenges, enablers, facilitators, and benefits arising from the integration of Industry 4.0 technologies and established management practices. By actively exploring these dimensions, our research goes beyond isolated assessments, filling the void in existing studies by offering a holistic exploration of the effects of Industry 4.0 technologies and management practices. This concerted effort to unravel the intricate dynamics and synergies contributes to building a robust foundation for strategic decision-making, which is essential for organizations seeking to enhance the sustainability and performance of their supply chains in the face of modern complexities. In essence, our study aspires to not only address the research gap but to also provide actionable insights that pave the way for a more sustainable and resilient global supply chain ecosystem.

## 3 Research methodology

Our research strategy is intricately linked to the specific goals we aim to achieve in this study, as emphasized by prior research [46]. Recognizing this connection, we have opted to utilize a thematic review approach. Thematic reviews offer a valuable degree of flexibility, making them well-suited for our objective. This method empowers us to engage in an in-depth exploration of existing ideas, uncover recurring themes woven across various studies, and ultimately synthesize the cumulative knowledge within the field of supply chain management [47,48]. This aligns with the research methodologies employed in similar studies within the domain of supply chain management. For instance, Behl and Dutta [49], conducted a thematic review of 362 papers published between 2011 and 2017 within humanitarian supply chain management, ultimately concluding that these papers could be categorized into nine key themes.

Similarly, Dixit and Dutta [50] adopted a thematic review approach to investigate the healthcare supply chain in disaster scenarios. Their research aimed to identify the main challenges faced in this context and propose future research directions to address them. Additionally, Zahraee et al. [51] conducted a systematic review of 300 studies focusing on biomass supply chain (BSC) modeling and optimization. Their thematic review approach allowed them to identify key challenges and propose future research directions within this specific domain of supply chain management. Likewise, Jia et al. [52] conducted a thematic review of 55 articles to identify key themes and topics related to soybean supply chain sustainability. This review culminated in the development of a novel conceptual framework to aid in managing sustainability efforts within this particular supply chain.

Furthermore, Younis et al. [53] employed a thematic review to investigate the application of artificial intelligence and machine learning within supply chains. Their findings indicated that the adoption of AI and ML technologies could potentially mitigate the bullwhip effect, thereby contributing to enhanced supply chain efficiency and responsiveness. Finally, Younis et al. [54] most recently utilized a thematic review approach to explore the adoption of blockchain technology in supply chain management. Their research aimed to identify the potential benefits, challenges, and critical factors influencing the implementation of this technology across various supply chain applications.

In our case, we will leverage the thematic review methodology to analyze relevant research articles found within academic databases such as Scopus and Google Scholar. By employing this approach, we aim to gain a comprehensive understanding of the current state of knowledge within our chosen field of study. This understanding will then serve as a valuable foundation for guiding our future research endeavors.

Consequently, the purpose of this study is to conduct a thematic literature assessment of the most relevant papers that addressed the impact of BC, IoT, BD, Lean, JIT, TQM, and manufacturer-led decentralization on sustainability in SC. The reason why the focus is on these specific technologies and such management practices is because it is believed that in the pursuit of sustainable supply chains, it is essential to study the impact of technologies such as AI, ML, BD, and IoT to unlock their potential for optimizing resource utilization, facilitating real-time monitoring, and promoting proactive sustainability measures. These innovative tools provide insights for eco-friendly product design, risk mitigation, and effective supply chain operations. Concurrently, it is essential to investigate management practices such as lean, JIT, TQM, and manufacturer-led decentralization. Integrating these practices facilitates a culture of sustainability by streamlining operations, enhancing stakeholder collaboration, and empowering localized decision-making. By comprehensively examining both technologies and management practices, businesses can tailor sustainable strategies, strike a balance between economic and environmental objectives, and drive long-term commitment to sustainability across the supply chain.

### 3.1 Research protocol

Publications in Scopus and Google Scholar were used to search for all related studies published up to December 31st, 2022. These databases were utilized because of the breadth with which articles could be searched, as well as the ease with which they could be categorized by publication type, author name, number of citations, and date of publication [17]. A similar research methodology has been adopted by many researchers [36,[51], [55], [56]]. The search was initially carried out with the combination of “sustainability and “supply chain” only within the title of the publication to remain focused, narrow down the results, and increase the relevance of the articles.

In Table 4 below, this study outlines the search process conducted using Scopus and Google Scholar to identify relevant studies on supply chain sustainability. The search, executed on February 28th, 2023, covered articles published until December 31st, 2022. The search criteria for this review included the keywords "sustainability" and "supply chain" in the titles of publications. This approach is consistent with the methodology adopted by many researchers, including Younis et al. [54].

**Table 4:** Material collection process.

Step		Excluded	Included
Identification	Initial Search-title contains "sustainability" and "supply chain"	–	1112
	Remove duplicates	70	1042
Screening	Exclude Book Chapters, Reviews, Notes & Conference Papers	222	820
	Exclude inaccessible publications	160	660
Eligibility	Exclude non-English papers	15	645
	Limit to published papers	100	545
Finalized	Focus on Business, Management & Social Science	273	322
	Exclude out-of-scope papers	272	**50**

Initially, 1112 studies with sustainability and supply chain-related terms in their titles were retrieved. Subsequently, 70 duplicate studies were removed, leaving 1042 papers. The exclusion of book chapters, reviews, editorials, and brief survey records resulted in 820 studies for further evaluation. During the screening phase, 160 inaccessible articles were removed, along with 15 articles in languages other than English. Additionally, 100 articles were in the pre-publication stage, and 273 did not fall under business management or social sciences, leading to their removal. After this rigorous screening, the remaining 272 studies underwent detailed analysis. Of these, 134 were found outside the scope of this review and excluded. Consequently, the final selection comprised 50 peer-reviewed journal articles that met the specific inclusion criteria, with a particular focus on Industry 4.0 technologies such as the Internet of Things (IoT), blockchain, and big data, and management practices like total quality management (TQM), just-in-time (JIT), vendor-managed inventory (VMI), lean practices, and manufacturer-led decentralized systems.

## 4 Discussion and analysis

In the following discussion section, we delve into a comprehensive examination and review of key management practices and leading Industry 4.0 technologies within the realm of supply chain sustainability. While previous research has often focused on isolated aspects, such as individual management practices or specific Industry 4.0 technologies, our study takes a holistic approach. We consider a wide array of management practices, including total quality management, just-in-time, vendor-managed inventory, lean, and manufacturer-led decentralized systems, alongside cutting-edge Industry 4.0 technologies like blockchain, the Internet of Things (IoT), and big data. By exploring these diverse elements in conjunction, we aim to provide a more nuanced understanding of their impact on supply chain sustainability.

The review will include both a descriptive analysis and an in-depth examination of the chosen papers. It starts off with a statistical breakdown of the selected papers, including a summary of key findings, distribution by the research techniques employed, data collection tool chosen, theoretical lenses drawn, journals targeted, contexts investigated, and industries covered. The in-depth examination phase shall include a discussion of publications that have been frequently cited, the performance dimensions addressed, the sustainability enablers identified, the sustainability challenges captured, and the sustainability benefits reported.

### 4.1 Descriptive analysis

#### 4.1.1 Theoretical lenses

**Fig. 1:**
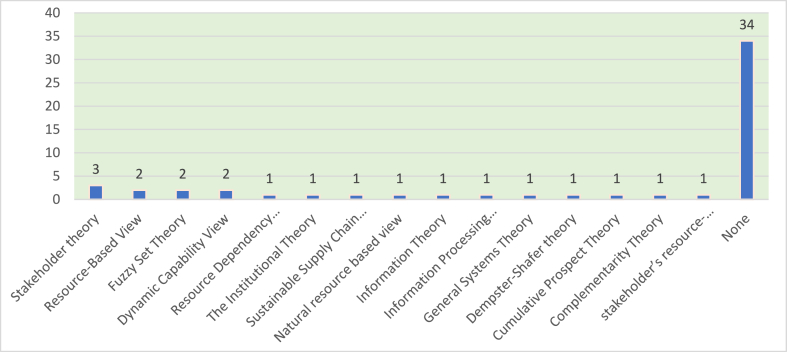
Distribution by theories used.

The analysis revealed that 32 % of the articles were based on a certain theoretical grounding, while 68 % opted to pursue a theory-building approach. The potential explanation as to why some authors didn't use certain theoretical grounding could be to increase the scope of their studies, conduct interdisciplinary research, or opt for a theory-building rather than theory-testing approach. Among the most commonly used theories are the stakeholder theory and the resource-based view, which were applied by authors three times and two times, respectively. To elaborate, stakeholder theory and resource-based views were used by Yildiz Çankaya and Sezen [57], who sought to find the impact of eight green supply chain management practices on the organizational sustainable performance of manufacturing companies in Turkey. Additionally, Mani et al. [58] employed insights from stakeholder theory to investigate the relationship between socially sustainable practices and supply chain performance within the Indian context. Finally, Govindan et al. [59] draw on stakeholder theory to test the impact of various sustainable practices on firm performance. Furthermore, the resource-based view was selected by Karaosman et al. [60] as their sole theoretical lens to help explain how the integration of sustainability practices across many tiers in two Italian luxury supply chains that produce leather footwear and apparel will improve the chain's overall sustainable performance. Fig. 1 below displays the frequency of theories used, while Table 5 displays the different theories applied along with the targeted performance dimension.

**Table 5:** Theories adopted.

Authors	Theory	Performance Dimension
Agyabeng-Mensah et al. [61]	Resource Dependency Theory	Economic
Bag et al. [62]	Information Theory and Dynamic Capability View	Sustainable
Baghizadehet al. [63]	Fuzzy Set Theory	Sustainable
Bappy et al. [64]	Dempster-Shafer theory	Sustainable
Bastas and Liyanage [65]	Sustainable Supply Chain Quality Management Theory	Sustainable
Chowdhury and Quaddus [66]	Dynamic Capability Theory	Sustainable
Govindan et al. [59]	Natural resource-based view and Stakeholder theory	Sustainable
Green et al. [28]	Complementarity Theory	Environmental
Hannibal and Kauppi [67]	Information Processing Theory	Sustainable
Karaosman et al. [60]	Resource-Based View	Economic and Environmental
Karmaker et al. [68]	Fuzzy Set Theory	Sustainable
Khan et al. [69]	Cumulative Prospect Theory	Social
León-Bravo et al. [70]	The Institutional Theory	Sustainable
Mani et al. [58]	Stakeholder's resource-based view	Social
Orji and Liu [71]	General Systems Theory	Sustainable
Yildiz Çankaya and Sezen [57]	Resource-Based View and Stakeholder Theory	Sustainable

#### 4.1.2 Publication by context

**Fig. 2:**
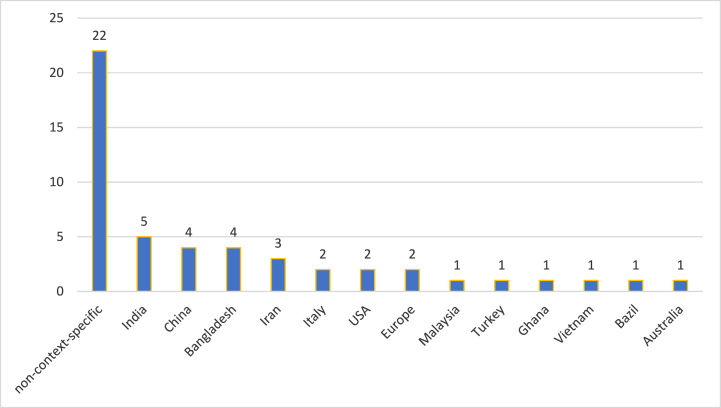
Distribution by context.

Generally speaking, 56 % of the articles were contextually driven, while 44 % didn't focus on a particular region or country. To explain, five studies examined the Indian context as one of the most important emerging economies. On the other hand, China and Bangladesh had 4 studies each, while the Iranian context was investigated by 3 studies. Next come Italy, the USA, and Europe, with two articles each. Lastly, six countries had one article each, including Malaysia, Turkey, Ghana, Vietnam, Brazil, and Australia. Fig. 2 below shows the distribution by context.

#### 4.1.3 Findings of key studies

While many of the selected papers provided a general review, a good number novelize by proposing a framework, developing a model, or suggesting a new methodology for approaching supply chain sustainability. These selected papers often lay the groundwork for further research, offering critical insights, innovative conceptualizations, and methodological advancements.

For example, Venkatesh et al. [36] developed a system architecture to combine blockchain, IoT, and big data analytics for social sustainability tracking. Similarly, Govindan et al. [59] suggested a framework for social sustainability that integrates drivers, issues, barriers, conflicts, practices, and performances. Moreover, Jabbour et al. [72] suggested a technique for expanding the amount of knowledge on sustainable multi-tiered supply chains. Additionally, Bappy et al. [64], Krishnan et al. [73], and Cabernard et al. [74] proposed a framework for measuring sustainability efficiency. On the other hand, Gardner et al. [75] and Hosseini-Motlagh et al. [76] presented a model to improve sustainability implementations within organizations. At the same time, Xu et al. [77] proposed a framework for assessing sustainability risks. Besides, and from a supply chain measurement perspective, D'Eusanio et al. [78] as well as Negri et al. [79] significantly contributed to the earlier development of a toolbox to measure sustainability performance and the later development of sustainability related KPIs. Finally, Zavala-Alcívar et al. [80] and Baghizadeh [63] developed a model to manage resilience and improve sustainability. Table 6 below summarizes key findings for the selected papers.

**Table 6:** Summary of key findings.

Authors	Key findings
Venkatesh et al. [36]	Developed a system architecture to integrate blockchain technology, IoT, and big data analytics for social sustainability traceability.
Govindan et al. [59]	Proposed a framework of social sustainability linking drivers, issues, barriers, tensions, practices, and performances.
Jabbour et al. [72]	Suggested a method to develop the body of knowledge in multi-tier supply chain sustainability.
Bappy et al. [64]	Proposed a framework for measuring sustainability efficiency
Bag et al. [62]	Proposed a framework for sustainability enablers
Yildiz Çankaya and Sezen [57]	Investigated the impact of various GSCM practices on different performance dimensions.
Gardner et al. [75]	Presented a model to improve sustainability.
Krishnan et al. [73]	Proposed a framework for measuring sustainability efficiency
Xu et al. [77]	Proposed a framework for assessing sustainability risks
D'Eusanio et al. [78]	Developed a toolbox to measure sustainability performance
Hosseini-Motlagh et al. [76]	Presented a model to improve sustainability.
Zavala-Alcívar et al. [80]	Developed a conceptual framework to manage resilience and improve sustainability.
Kalantary et al. [81]	Introduced a model to assess the sustainability of supply chains over multiple periods.
Cabernard et al. [74]	Proposed a framework for measuring sustainability efficiency
Neri et al. [82]	Developed sustainability-related KPIs
Hannibal and Kauppi [67]	Highlighted the challenges of sustainability.
Baghizadeh [63]	Presented a model to minimize costs and maximize social benefits.

#### 4.1.4 Publication by industry

The analysis revealed that 13 researchers did not base their studies on a certain industry, while twelve specific industries were targeted by the remaining 33 articles. This could be explained by claiming that similar sustainability concerns, such as lowering greenhouse gas emissions, eliminating waste, and protecting natural resources, affect many different industries.

**Fig. 3:**
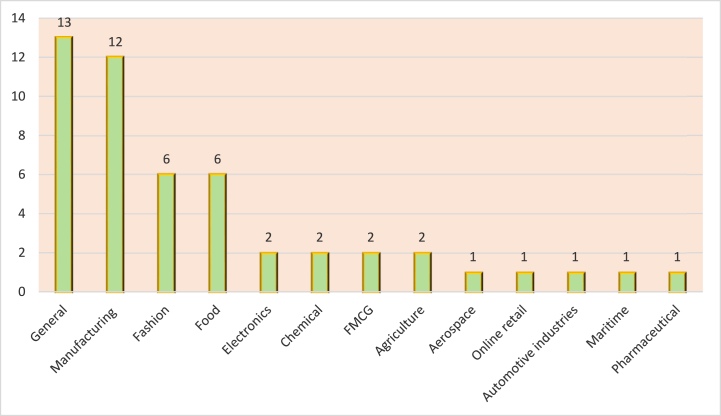
Distribution by industry.

Based on Fig. 3 below, which shows the distribution of articles by industry, the manufacturing sector came in first with 12 articles, indicating that this sector is among the important domains for sustainability. Evidently, and within the US, approximately 23 % of total U.S. greenhouse gas emissions resulted from manufacturing activities (Environmental Protection Agency, 2019).

Among the studies that target the manufacturing industry are Gupta et al. [83]; Yildiz Çankaya and Sezen [57]; Bai et al. [84]; Agyabeng-Mensah et al. [61]; Green et al. [28]; Karmaker et al. [68]; Sharma et al. [85]; Silva and Figueiredo [86]; Mani et al. [58]; Govindan et al. [59]; Jadhav et al. [87]; Baghizadeh [63]. The next two important sectors are the fashion and food industries, with six articles each. Amongst the important studies within the food sector is the work of Malak-Rawlikowska et al. [88]; Vittersø et al. [89]; Jia et al. [52]; León-Bravo et al. [70]; Sharma et al. [90]; and Krishnan et al. [73]. Similarly, below are the highly cited articles that targeted the fashion industry: Karaosman et al. [60]; Munny et al. [91]; Chowdhury and Quaddus [66]; Hannibal and Kauppi [67]; Mejías et al. [92]; Xu et al. [77]. The electronics industry, chemicals, FMCG, and agriculture were targeted by two studies each. Finally, one article has focused on each of the following industries: aerospace, online retail, pharmaceutical, maritime, and automotive.

#### 4.1.5 Publication by journal

The 50 articles were published in 25 reputable journals, of which 20 % appeared in the Journal of Cleaner Production. Another important journal is the International Journal of Production Economics, with nine articles. Next comes Sustainable Production and Consumption, with 4 articles. Table 7 below shows the distribution by journal.

**Table 7:** Distribution by journals.

Name of the Journal	No. of papers	Name of the Journal	No. of papers
Journal of Cleaner Production	10	IEEE Transactions on Systems, Man, and Cybernetics: Systems	1
International Journal of Production Economics	9	Environmental Impact Assessment Review	1
Sustainable Production and Consumption	4	Benchmarking: An International Journal	1
Sustainability (Switzerland)	3	Business Strategy and the Environment	1
International Journal of Production Research	2	Management Decision	1
Computers and Industrial Engineering	2	Mathematical Problems in Engineering	1
Journal of Manufacturing Technology Management	2	Operations Management Research	1
Journal of Business Research	1	Resources, Conservation and Recycling	1
International Journal of Logistics Management	1	Operations Research for Health Care	1
International Journal of Operations and Production Management	1	Science of the Total Environment	1
International Journal of Logistics Management	1	Robotics and Computer-Integrated Manufacturing	1
Information Sciences	1	Transportation Research Part E: Logistics and Transportation Review	1

#### 4.1.6 Research methods and data gathering tools

According to the findings of the analysis, roughly 78 % of the studies relied on a qualitative research approach to reach their research objectives. This approach may involve conducting interviews, analyzing expert opinions, conducting content analysis, performing literature reviews, or using case studies.

**Fig. 4:**
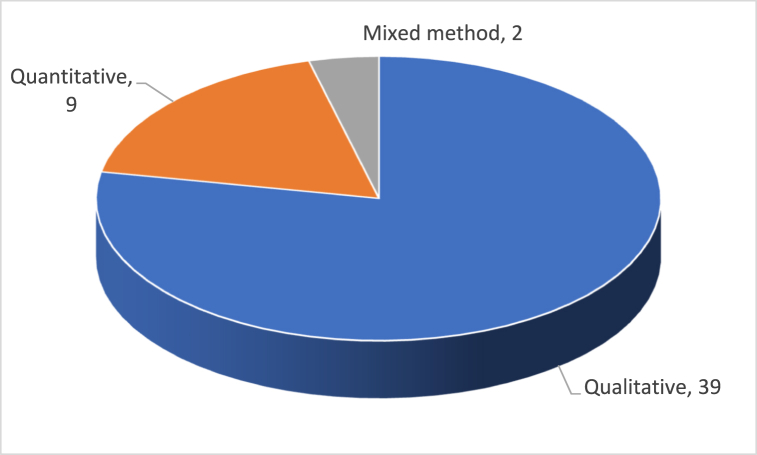
Distribution by research method.

**Fig. 5:**
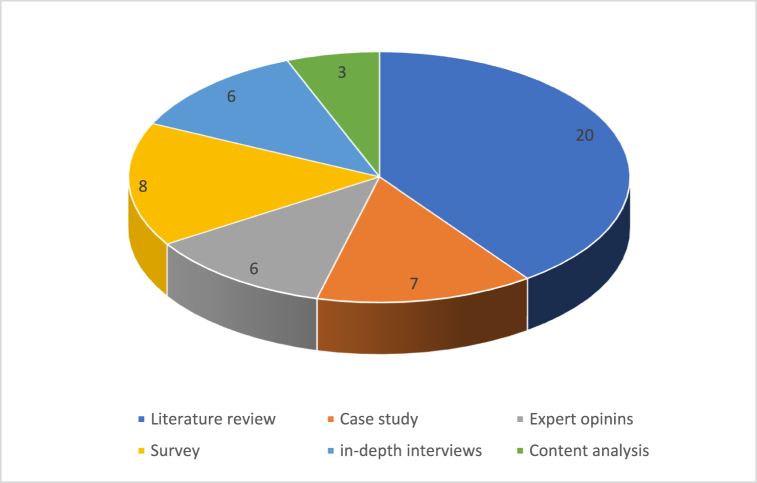
Distribution by data gathering tool.

18 % of the studies used quantitative research methods, employing a variety of statistical techniques to confirm the authors' hypotheses. Finally, only two of the publications, or 4 %, made use of the methodology known as triangulation, which combines qualitative and quantitative research methods in order to evaluate and validate the authors' hypotheses. The two pie charts in Fig. 4, Fig. 5 below visualize the different research and data gathering tools used in these fifty publications.

The descriptive analysis of the selected articles provides valuable insights into the landscape of research on supply chain sustainability, shedding light on various theoretical frameworks, methodological approaches, contextual factors, and industry focuses. Notably, a significant portion of the studies employed theory-building approaches, highlighting the importance of conceptualizing new frameworks and models to address sustainability challenges effectively. Stakeholder theory and the resource-based view emerged as the most commonly utilized theoretical lenses, offering robust frameworks for examining the relationship between sustainability practices and organizational performance. Moreover, several studies proposed innovative frameworks, models, and methodologies, paving the way for further research and practical applications in the field. These contributions include the development of system architectures, sustainability frameworks, measurement tools, and resilience models, offering actionable insights for supply chain practitioners, policymakers, and managers. Furthermore, the analysis revealed a diverse range of contextual focuses, with the manufacturing sector receiving significant attention due to its substantial environmental footprint. Other key industries targeted include fashion, food, electronics, chemicals, FMCG, and agriculture, reflecting the broad scope of sustainability concerns across different sectors. Overall, this comprehensive analysis underscores the importance of continued research efforts to address sustainability challenges and drive positive change within the global supply chain ecosystem.

### 4.2 In-depth examination of the chosen papers

#### 4.2.1 Highly cited articles

The cited studies in the literature review exhibit varying degrees of scholarly attention, with the highest-cited study garnering an impressive 239 citations, underscoring its significant impact and influence within the research community. In contrast, the least-cited study received 11 citations, indicating a comparatively lower level of recognition. For the purpose of this study, we will focus our discussion on the ten highly cited papers, as they represent a substantial body of research that has resonated prominently in the field, offering valuable insights and perspectives on the topic of supply chain sustainability.

The analysis reveals that the study by Bag et al. [62] is one of the most often cited works, which may be related to the authors' framework-based proposal of the key enablers influencing supply chain sustainability. The second notable study in terms of citations is Sarkis's [93] investigation of the impact of COVID-19 on the environmental sustainability of companies. The author drew the conclusion that the COVID-19 pandemic resulted in short-term environmental sustainability but that the long-term implications are still undetermined and require further study. The same results were coined recently by Younis et al. [55], who concluded that COVID-19 created many opportunities for supply chains in general and for certain industries in particular, including improved environmental sustainability. Another highly cited publication is the study of Yildiz Çankaya and Sezen [57], in which the authors employed a survey within the Turkish context to investigate how the three aspects of corporate sustainability; economic, environmental, and social are affected by the eight elements of a green supply chain management strategy: green manufacturing, green distribution, green packaging, green marketing, internal environmental management, and investment recovery.

Furthermore, the investigation by Gardner et al. [75] is another frequently cited work. This is because the authors suggested a typology to separate the different types of supply chain data that are needed to improve sustainability governance and to show a number of major flaws and biases in the way information systems are set up right now. Yet another crucial study that received a good number of citations is the research of Karmaker et al. [68]. The authors conducted research using the opinions of industry professionals and discovered that financial help from both the government and the partners in the supply chain is essential to addressing the immediate impact that COVID-19 is having on supply chain sustainability. They also found that for supply chains to be sustainable over the long run, the adoption of policies that take into consideration health protocols and automation is vital. In addition, the study by Venkatesh et al. [36] is another paper that has received a lot of citations. It might be as a result that the authors created a system architecture to integrate and apply blockchain, IoT, and big data analytics for social sustainability traceability in supply chains while examining the implementation costs and difficulties. By the same token, Green et al. [28] are among the most important studies in this domain. The authors aimed at conducting an empirical study to determine how JIT, TQM, and green supply chain techniques interact to complement one another's impact on environmental performance, concluding that JIT and TQM improve environmental performance when used together. Equally, the study by Govindan et al. [59] is on the list of the most referenced papers. It could be due to the fact that the authors provided a theoretical structure for social sustainability that involves interconnections between causes and effects, obstacles and solutions, stresses, and performances.

**Fig. 6:**
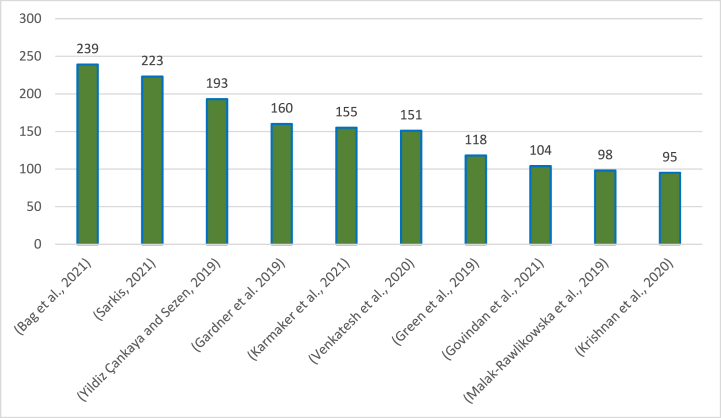
The 10 highly cited papers (as of February 28, 2023).

Similarly, the study by Malak-Rawlikowska et al. [88] is another widely-cited article. The significance of this study arises from the fact that it surveyed 208 food producers within 7 European countries and concluded that "longer" supply chains have lower environmental impacts per unit of production, as assessed by food miles and carbon footprint. Finally, Krishnan et al. [73] is among the publications with the most citations owing to the fact that the authors developed a plan to improve the mango food supply chain's operational and resource inefficiency. Fig. 6 summarized the distribution of the 10 highly cited papers. Fig. 6 summarizes the distribution of the 10 highly cited papers.

#### 4.2.2 Performance dimension

The 50 selected articles tackled several performance dimensions. Below are the primary dimensions addressed.

##### 4.2.2.1 Sustainable performance

The analysis revealed that 60 % of the studies investigated the sustainable performance of the organization, which is the integration of environmental, economic, and social dimensions. An example of highly cited papers that targeted sustainable performance is the work of Bag et al. [62]; Yildiz Çankaya and Sezen [57]; Gardner et al. [75]; Karmaker et al. [68]; Gupta et al. [83]; Sharma et al. [90]; Xu et al. [77]; D'Eusanio et al. [78]; Govindan et al. [59].

##### 4.2.2.2 Social performance

As far as social performance is concerned, the study shows that 16 % of the papers examined the social performance of the organization as one of the three important pillars of sustainability. The studies that were socially focused are: Venkatesh et al. [36]; Govindan et al. [59]; Mani et al. [58]; Hosseini-Motlagh et al. [76]; Jadhav et al. [87]; Munny et al. [91]; Vittersø et al. [89]; Khan et al. [69].

##### 4.2.2.3 Environmental performance

One of the cornerstones of sustainability is environmental performance. Accordingly, next in terms of research attention was the topic of environmental performance, with seven studies addressing it. The studies include Sarkis [93]; Green et al. [28]; Malak-Rawlikowska et al. [88]; Jadhav et al. [87]; Jia et al. [52]; Karaosman et al. [60]; Cabernard et al. [74]; and Suhi et al. [94].

##### 4.2.2.4 Economic performance

Economic performance is another important aspect of sustainability; hence, it was covered by four studies, which include the work of Lai et al. [95]; et al. [51]; Shi et al. [96]; and Agyabeng-Mensah et al. [61].

##### 4.2.2.5 Operational performance

**Fig. 7:**
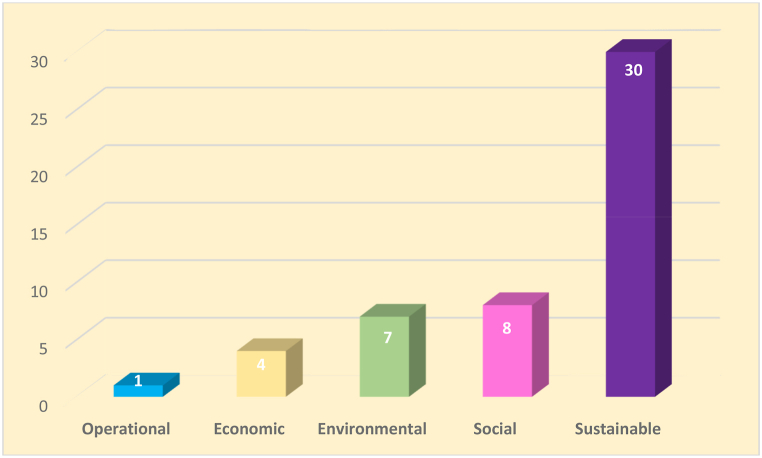
Distribution by performance dimension.

The analysis shows that operational performance is underrepresented, as only the study of Krishnan et al. [73] addressed this dimension. Fig. 7 shows the distribution by performance dimension.

#### 4.2.3 Challenges of sustainability

The authors of the selected papers reported a number of sustainability challenges in their analysis. These challenges include a lack of employees’ knowledge and awareness [90], information asymmetries between supply chain actors and stakeholders [67], a lack of performance measurement systems [79], a lack of sustainability techniques [90]; a lack of technology and poor government policies [[90], [97], [98], [99], [100]]. Table 8 below displays the challenges of sustainability.

**Table 8:** challenges of sustainability.

No.	Challenges	Authors
1	Information asymmetries between supply chain actors and stakeholders.	Hannibal and Kauppi [67]
2	Lack of employees' knowledge and awareness.	Sharma et al. [85]
3	Lack of performance measurement systems.	Negri et al. [79]
4	Lack of sustainability techniques.	Sharma et al. [90]
5	Lack of technology.	Sharma et al. [90]
6	Poor government policies.	Sharma et al. [90]

#### 4.2.4 Enablers of sustainability

While a number of scholars have noted difficulties in implementing sustainable practices, others have discovered a wealth of enabling factors that push businesses in that direction. Enabling factors mainly evolve around the regulatory and external environment, workplace health and safety, and financial and supply chain support. Examples of such enabling factors include: government regulations' and ‘conducive working conditions [71]; [101,102], government subsidies [96], workplace health and safety practices [91], financial support from the government as well as from the supply chain partners [68], openness and transparency-based relationships with the external environment [69], forecast sharing [95,103], and increased environmental tax rate [96,[104], [105], [106], [107]]. Table 9 below highlights the reported sustainability enablers.

**Table 9:** sustainability enablers.

No.	Enablers	Authors
1	Government regulations’ and ‘Conducive working conditions.	Orji and Liu [71]
2	Workplace health and safety practices.	Munny et al. [91]
3	Openness and transparency-based relationships with the external environment.	Khan et al. [69]
4	Financial support from the government as well as from the supply chain partners.	Karmaker et al. [68]
5	Forecast sharing.	Lai et al. [95]
6	Government subsidies.	Shi et al. [96]
7	Increased environmental tax rate.	Shi et al. [96]

#### 4.2.5 Benefits of sustainability

The investigation identified several advantages related to sustainability practice implementation. To mention a few: improve operational performance [72], improve waste management [94], improve workplace health and safety [91], reduce risks [66], improve firm performance [59], and improve supply chain performance [58]. Table 10 summarizes the benefits of sustainability.

**Table 10:** sustainability benefits.

No.	Benefits	Authors
1	Improve operational performance.	Mani et al. [58]
2	Improve waste management.	Suhi et al. [94]
3	Improves firm performance.	Govindan et al. [59]
4	Improves supply chain performance.	Mani et al. [58]
5	Reduces uncertainty in supply chain.	Hannibal and Kauppi [67]
6	Reduction of risks.	Chowdhury and Quaddus [66]
7	Improve workplace health and safety.	Munny et al. [91]

#### 4.2.6 Thematic analysis of the selected papers-sustainability facilitators

An in-depth investigation of this study found, among other things, that specific practices or technologies help with the successful implementation of sustainable measures. Lean principles, for example, are one type of approach that can help in successfully implementing sustainable measures, as reported by Ruiz-Benitez et al. [27] and Genc and De Giovanni [25]. Similarly, Green et al. [28] and Bastas and Liyanage [65] concluded that both JIT and TQM are directly and positively associated with sustainability practices. Another significant facilitator of sustainability is VMI, as concluded by Weraikat et al. [45]. Using a real-life case study from the pharmaceutical industry, the authors managed to prove that the implementation of VMI led to a significant reduction in the safety stock, which improved the sustainable performance of the organization.

##### **4.2.6.1** *Theme 1*: JIT, TQM and VMI role in sustainable supply chain

As per the interdisciplinary approach of the general systems theory, which seeks to understand complex systems by studying the patterns of relationships between their components, it can be claimed that the general systems theory can provide a unified framework on how lean, JIT, TQM, and VMI can facilitate the successful implementation of sustainability practices, and therefore, the following proposition has been created.

P1 The implementation of lean practices (JIT, TQM, and VMI) facilitates the adoption of sustainability-related practices and supports the general systems theory.

##### 4.2.6.2 Theme 2: blockchain, IoT, and big data role in sustainable supply chain

At the same time, the in-depth analysis revealed that a number of new technologies can play an important role in facilitating the implantation of sustainability practices. For example, Venkatesh et al. [36] developed a system architecture to integrate and utilize blockchain technology, IoT, and big data analytics for social sustainability traceability in supply chains, then analyzed the cost and difficulties associated with implementing the system. Furthermore, Park and Li [35] concluded that blockchain technology has the potential to improve supply chain sustainability performance. Henceforward, and drawing on the dynamic capability view, which states that businesses who are able to grow and improve their dynamic capabilities over time will be better equipped to adapt to shifting market circumstances and preserve their competitive edge, In order to do this, it is necessary to invest in the creation of new technologies, procedures, and business models, as well as cultivate a culture of innovation and ongoing learning. Accordingly, organizations that seek to remain competitively advantaged through sustainability shall also consider utilizing new technologies such as blockchain, IoT, and bid data to facilitate the adoption of sustainability practices. Therefore, the following proposition has been created.

P2 The adoption of new technologies such as blockchain, IoT, and big data facilitates implementing sustainability practices and complements the dynamic capability theory.

##### 4.2.6.3 Theme 3: A manufacturer-led decentralized system role in sustainable supply chain

Finally, the analysis shows that a manufacturer-led decentralized system, in which the manufacturer remains accountable for production but other parties, including suppliers, distributors, and even customers, may have input into and even direct inventory management, quality control, and distribution, can facilitate the adoption of sustainability practices, as evidenced by Bai et al. [84].

**Fig. 8:**
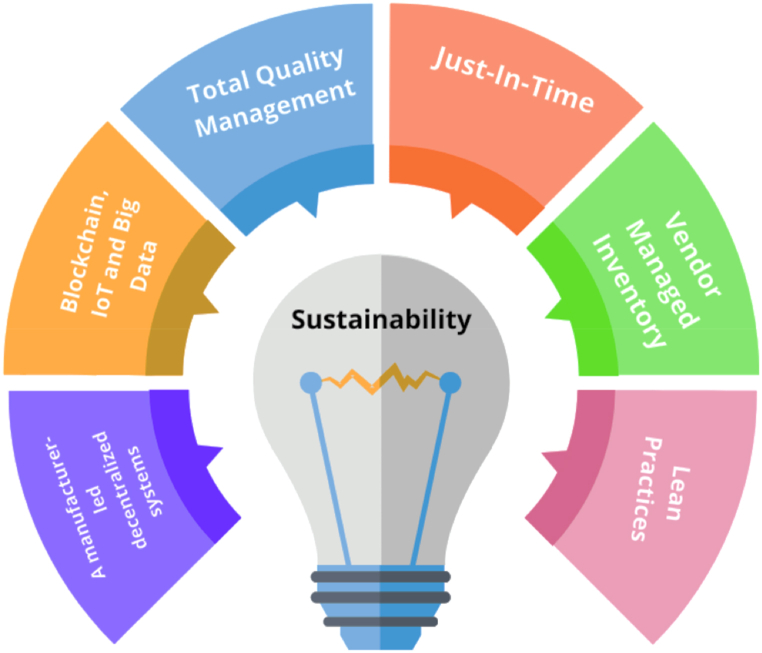
Facilitators of sustainability.

Correspondingly, a manufacturer can improve productivity, adaptability, and responsiveness to customer needs by decentralizing decision-making and control [84]. Yet, it also necessitates careful coordination and open lines of communication among the various parties involved to ensure that everyone is working toward the same end result as the manufacturer [87]. Moreover, openness and transparency-based relationships with the external environment can improve supply chain sustainability [69]; 86. The aforementioned argument is consistent with supply chain collaboration theory, which postulates that cooperation among supply chain members can lead to a competitive advantage. For that reason, the following proposition is reasonable to make:

P3. A manufacturer-led decentralized system can facilitate the adoption of sustainable practices leading to competitive advantage and complementing the resource-based view theory. Fig. 8 below highlights the main facilitators of sustainability.

The thematic review explored a diverse array of studies on supply chain sustainability, revealing a spectrum of scholarly attention and pivotal contributions within the field. Highly cited works by Bag et al. [62] Sarkis [93], and others provided robust frameworks addressing sustainability enablers and the repercussions of external factors such as COVID-19. Studies by Yildiz Çankaya and Sezen [57] illuminated the intricate interplay between green supply chain strategies and organizational sustainability. Environmental performance emerged as a central theme, with social and economic dimensions also receiving notable scrutiny. Common challenges identified in the review encompassed knowledge gaps and inadequate measurement systems, while enabling factors such as regulatory support and technological innovations were recognized. The synthesis of findings led to the formulation of three theoretical propositions aimed at bolstering sustainability practices, thereby emphasizing the crucial roles of management practices, new technologies, and decentralized decision-making structures. Overall, the thematic review underscored the multifaceted nature of sustainability challenges in supply chains and advocated for holistic approaches to address them effectively.

## 5 Conclusion

The substantial number of research papers addressing sustainability in supply chains stands in stark contrast to the relative dearth of studies investigating the specific impact of management practices and Industry 4.0 technologies on supply chain sustainability. While sustainability within supply chains has garnered considerable attention, there exists a notable gap in the literature when it comes to comprehensively exploring how management strategies and cutting-edge Industry 4.0 technologies intersect with and influence sustainability initiatives. By bridging this research gap, a more holistic understanding of how management practices and technology adoption can enhance supply chain sustainability is achieved, enabling organizations to make more informed decisions and contribute to a more responsible and resilient global supply chain ecosystem.

Accordingly, after a critical review and a meticulous analysis of 50 relevant and highly cited studies published in reputable journals, this research has elucidated the role that several management practices, such as lean principles, JIT, TQM, VMI, and a manufacturer-led decentralized system, as well as industry 4.0 technologies including blockchain, IoT, and big data, can play in the successful application of sustainable practices [28]; [27,45]. Three propositions have been developed to aid businesses in their pursuit of sustainability and competitive advantage. These propositions draw on general systems theory, the dynamic capability view, and supply chain collaboration theory. Organizations can improve their overall performance and competitiveness and get closer to sustainability if they consider the facilitators and propositions. In conclusion, this study significantly enhances our understanding of sustainability by not only identifying key factors such as challenges, enablers, benefits, and facilitators but also by shedding light on the predominant performance dimensions that sustainability can enhance. Furthermore, it emphasizes the pivotal role of technologies and effective management practices in elevating sustainability efforts to new heights. Thus, the current study contributes to the existing literature on supply chain sustainability by identifying key management practices and Industry 4.0 technologies that enhance sustainability initiatives. Through a comprehensive review and analysis of relevant studies, this research synthesizes theoretical insights and practical implications, offering valuable guidance for practitioners, policymakers, and managers. By developing novel theoretical propositions and highlighting practical pathways for sustainability practice adoption, this study provides a framework for advancing organizational performance, competitiveness, and sustainability within the global supply chain ecosystem.

### 5.1 Theoretical implications

Following a comprehensive analysis of the selected articles, several notable theoretical insights have emerged. To delve deeper into these findings, it is evident that various authors leveraged multiple theoretical frameworks to construct their arguments while assessing the impact of sustainability practices on five distinct performance dimensions: sustainability, environmental, social, economic, and operational. Furthermore, our in-depth review underscored a multitude of sustainability-related factors, meticulously categorized as challenges, enablers, benefits, and facilitators. These factors wield significant influence, potentially either bolstering or obstructing the implementation of sustainability practices. Notably, our research makes a substantial theoretical contribution through the development of novel theoretical propositions designed to enhance the adoption and integration of sustainability practices within diverse contexts.

### 5.2 Practical implications

The implications of this study's findings for practitioners, policymakers, and managers are substantial. For example, understanding the challenges that face sustainability practice implementation can help supply chain practitioners make the right decisions as to how to overcome such challenges by exploiting the available enablers, such as support from supply chain partners [68] and government subsidies [96,[108], [109], [110], [111], [112]]. Additionally, the study offers significant insights into the current state of research on supply chain sustainability, highlighting the key facilitators of sustainability practice adoption such as JIT and TQM [28]; 65, VMI [45], and lean principles [27]. In conclusion, the study offers significant theoretical and practical implications, and the findings have crucial consequences for supply chain practitioners, policymakers, and managers.

### 5.3 Challenges of existing studies and future prospects

Despite the burgeoning body of research addressing sustainability in supply chains, the literature reveals notable challenges. Existing studies often fall short in investigating the specific impact of management practices and Industry 4.0 technologies on supply chain sustainability comprehensively. The tendency to focus on isolated elements rather than exploring the intricate dynamics arising from the convergence of these factors poses a challenge. Furthermore, the theoretical frameworks employed in some studies may lack integration, hindering a holistic understanding of the multifaceted relationship between sustainability, management practices, and Industry 4.0 technologies. The gap between theoretical propositions and practical implementation also remains a challenge, requiring more nuanced insights to bridge the divide.

This research paves the way for significant prospects in advancing the understanding of supply chain sustainability. The critical review and meticulous analysis of 50 relevant and highly cited studies have uncovered promising prospects for the integration of management practices and Industry 4.0 technologies. The identified facilitators, such as lean principles, JIT, TQM, VMI, and a manufacturer-led decentralized system, offer tangible pathways for organizations to enhance their sustainable practices. The development of three theoretical propositions based on general systems theory, the dynamic capability view, and supply chain collaboration theory provides a theoretical foundation for future research endeavors. These propositions not only contribute to theoretical advancements but also offer practical avenues for businesses to improve overall performance, competitiveness, and sustainability by considering the identified facilitators.

In addition to the prospects identified within the selected studies, this research opens avenues for further exploration of theoretical insights. The emerging theoretical propositions provide a framework for future studies to delve deeper into the integration of sustainability practices across diverse contexts. Practical implications extend to supply chain practitioners, policymakers, and managers, offering actionable insights into overcoming challenges and leveraging facilitators for sustainability practice adoption. The findings not only contribute to the theoretical understanding of sustainability but also have significant consequences for decision-making in the practical realm of supply chain management.

## Availability of data and material

Authors confirm that all relevant data are included in this manuscript, and all sources are well cited.

## Ethical statements

All subjects gave their informed consent for inclusion before they participated in the study.

## Funding

This research received no external funding.

## CRediT authorship contribution statement

**Manaf Al-Okaily:** Writing – original draft, Supervision, Resources, Project administration, Methodology, Conceptualization. **Hassan Younis:** Writing – original draft, Visualization, Validation, Methodology, Formal analysis, Data curation. **Aws Al-Okaily:** Writing – review & editing, Methodology, Conceptualization.

## Declaration of competing interest

The authors declare the following financial interests/personal relationships which may be considered as potential competing interests:Manaf Al-Okaily reports a relationship with Jadara University that includes: board membership. Associate Editor in Heliyon Journal If there are other authors, they declare that they have no known competing financial interests or personal relationships that could have appeared to influence the work reported in this paper.

## References

[1] Aws A.L., Teoh A.P., Al-Okaily M. (2021). Towards business intelligence success measurement in an organization: a conceptual study. Journal of System and Management Sciences.

[2] Silva Minelle E., Marina D. Figueiredo (2020). Practicing sustainability for responsible business in supply chains. J. Clean. Prod..

[3] McDonough William, Braungart Michael (2010).

[4] Modak Nikunja Mohan, Sinha Sudipta, Kumar Ghosh Debabrata (2023). A review on remanufacturing, reuse, and recycling in supply chain—exploring the evolution of information technology over two decades. International Journal of Information Management Data Insights.

[5] Gong Mengfeng, Yuan Gao, Koh Lenny, Sutcliffe Charles, Cullen John (2019). The role of customer awareness in promoting firm sustainability and sustainable supply chain management. Int. J. Prod. Econ..

[6] Toktaş-Palut Peral (2022). Analyzing the effects of Industry 4.0 technologies and coordination on the sustainability of supply chains. Sustain. Prod. Consum..

[7] Badran A. (2023). Artificial intelligence between government and self-regulation policies: a theoretical approach. Hikama.

[8] Alflaieh M.T. (2022). Electronic fraud in the context of E-commerce under Jordanian legislation. Al-Zaytoonah University of Jordan Journal for Legal studies.

[9] Elkington John (1994). Towards the sustainable corporation: win-win-win business strategies for sustainable development. Calif. Manag. Rev..

[10] Younis Hassan, Sundarakani Balan, Vel Prakash (2016). The impact of implementing green supply chain management practices on corporate performance. Compet. Rev..

[11] Carter Craig R., Liane Easton P. (2011). Sustainable supply chain management: evolution and future directions. Int. J. Phys. Distrib. Logist. Manag..

[12] Beamon Benita M. (1999). Designing the green supply chain. Logist. Inf. Manag..

[13] Chow Garland, Heaver Trevor D. (2018). Global Logistics and Distribution Planning.

[14] Mentzer John T., DeWitt William, Keebler James S., Min Soonhong, Nix Nancy W., Smith Carlo D., Zacharia Zach G. (2001). Defining supply chain management. J. Bus. Logist..

[15] Shapiro J.F. (2001). Modeling and IT perspectives on supply chain integration. Inf. Syst. Front.

[16] Pienaar W. (2009). Southern Africa.

[17] Wu Lifang, Yue Xiaohang, Jin Alan, Yen David C. (2016). Smart supply chain management: a review and implications for future research. Int. J. Logist. Manag..

[18] Dutta D.K., Hora M. (2017). From invention success to commercialization success: technology ventures and the benefits of upstream and downstream supply‐chain alliances. J. Small Bus. Manag..

[19] Zhao Dangzhi, Strotmann Andreas (2015).

[20] Al-Okaily M. (2023). The influence of esatisfaction on users' eloyalty toward ewallet payment apps: a mediatedmoderated model. Int. J. Emerg. Mark..

[21] Al-Okaily M. (2023).

[22] Teoh A.P., Al-Okaily M. (2023). Evaluation of data analytics-oriented business intelligence technology effectiveness: an enterprise-level analysis. Bus. Process Manag. J..

[23] Al-Okaily M. (2022). Toward an integrated model for the antecedents and consequences of AIS usage at the organizational level. EuroMed J. Bus..

[24] Al-Okaily M., Al-Okaily A. (2022). An empirical assessment of enterprise information systems success in a developing country: the Jordanian experience. The TQM Journal.

[25] Genc Talat S., Pietro De Giovanni (2020). Closed-loop supply chain games with innovation-led lean programs and sustainability. Int. J. Prod. Econ..

[26] Younis H. (2019). Embedding Culture and Quality for High Performing Organizations.

[27] Ruiz-Benitez Rocio, López Cristina, Real Juan C. (2019). Achieving sustainability through the lean and resilient management of the supply chain. Int. J. Phys. Distrib. Logist. Manag..

[28] Green Kenneth W., Anthony Inman R., Sower Victor E., Zelbst Pamela J. (2019). Impact of JIT, TQM and green supply chain practices on environmental sustainability. J. Manuf. Technol. Manag..

[29] Teoh A.P., Al-Okaily M., Iranmanesh M., Al-Betar M.A. (2023). The efficiency measurement of business intelligence systems in the big data-driven economy: a multidimensional model. Information Discovery and Delivery.

[30] Al-Kofahi M., Shiyyab F.S., Al-Okaily A. (2023). Determinants of user satisfaction with financial information systems in the digital transformation era: insights from emerging markets. Global Knowledge. Memory and Communication.

[31] Al-Okaily M., Al-Okaily A. (2024). Financial data modeling: an analysis of factors influencing big data analytics-driven financial decision quality. J. Model. Manag..

[32] Faguet J. (2023). Decentralization and governance. Hikama.

[33] Al-Shahrani H. (2023). Examining the extent of faculty members acceptance and readiness on shifting to the E-learning system during the COVID-19 pandemic through the lens of GETAMEL. Journal of Education /Al Mejlh Altrbwyh.

[34] Abd Rahman M.S., Ali A., Abu-Shanab E., Masa’deh R. (2023). An empirical investigation on acceptance of mobile payment system services in Jordan: extending UTAUT2 model with security and privacy. Int. J. Bus. Inf. Syst..

[35] Park Arim, Huan Li (2021). The effect of blockchain technology on supply chain sustainability performances. Sustainability.

[36] Venkatesh V.G., Kang K., Wang B., Zhong R.Y., Zhang A. (2020). System architecture for blockchain-based transparency of supply chain social sustainability. Robot. Comput. Integrated Manuf..

[37] Liu L., Song W., Liu Y. (2023). Leveraging digital capabilities toward a circular economy: reinforcing sustainable supply chain management with Industry 4.0 technologies. Comput. Ind. Eng..

[38] Alshammry A. (2023). The degree of enabling distance E-learning for first graders from the language competencies necessary for them. Journal of Education /Al Mejlh Altrbwyh.

[39] Al-Okaily A., Al-Okaily M., Teoh A.P. (2023). Evaluating ERP systems success: evidence from Jordanian firms in the age of the digital business. VINE Journal of Information and Knowledge Management Systems.

[40] Al-Okaily A., Al-Okaily M., Teoh A.P., Al-Debei M. (2023). An empirical study on data warehouse systems effectiveness: the case of Jordanian banks in the business intelligence era. EuroMed J. Bus..

[41] Magatef S., Al-Okaily A., Shiyyab F.S. (2024). Exploring the factors that influence academic performance in Jordanian higher education institutions. Heliyon.

[42] Al-Majali D., Al-Okaily A., Majali T. (2023). Blockchain technology and its applications in digital accounting systems: insights from Jordanian context. J. Financ. Report. Account..

[43] Al-Fulaij Shaikha, Al-Qudsi Sulayman, Arman Hussam, Alawadhi Ahmad S. (2022). The potential role of exports-imports in income diversification and sustainable development of Kuwait. Arab Journal of Administrative Sciences.

[44] Patidar A., Sharma M., Agrawal R., Sangwan K.S. (2022). Supply chain resilience and its key performance indicators: an evaluation under Industry 4.0 and sustainability perspective. Manag. Environ. Qual. Int. J..

[45] Weraikat D., Zanjani M.K., Lehoux N. (2019). Improving sustainability in a two-level pharmaceutical supply chain through a Vendor-Managed Inventory system. Operations Research for Health Care.

[46] Almarri A., Elayah M. (2024). Financial sustainability of Qatar's third sector through direct public investment. Hikama.

[47] Hjij H. (2023). Digital social networks challenges to classical political culture theory. Siyasat Arabiya.

[48] Alrai A. (2023). Investigating and inferring crimes through artificial intelligence systems. Al-Zaytoonah University of Jordan Journal for Legal Studies.

[49] Behl A., Dutta P. (2019). Humanitarian supply chain management: a thematic literature review and future directions of research. Ann. Oper. Res..

[50] Dixit A., Dutta P. (2023). Thematic review of healthcare supply chain in disasters with challenges and future research directions. Int. J. Disaster Risk Reduc..

[51] Zahraee S.M., Golroudbary S.R., Shiwakoti N., Kraslawski A., Stasinopoulos P. (2019). An investigation of the environmental sustainability of palm biomass supply chains via dynamic simulation modeling: a case of Malaysia. J. Clean. Prod..

[52] Jia Fu, Zuluaga-Cardona Laura, Bailey Adrian, Rueda Ximena (2018). Sustainable supply chain management in developing countries: an analysis of the literature. J. Clean. Prod..

[53] Younis H., Wuni I.Y. (2023). Application of industry 4.0 enablers in supply chain management: scientometric analysis and critical review. Heliyon.

[54] Younis H., Bwaliez O.M., Al-Okaily M., Tanveer M.I. (2024). Revolutionizing supply chain management: a critical meta-analysis of empowerment and constraint factors in blockchain technology adoption. Bus. Process Manag. J..

[55] Younis Hassan, Alsharairi Malek, Younes Hammad, Sundarakani Balan (2023). The impact of COVID-19 on supply chains: systematic review and future research directions. Operational Research.

[56] Munny Azmina Akter, Ali Syed Mithun, Kabir Golam, Abdul Moktadir Md, Rahman Towfique, Mahtab Zuhayer (2019). Enablers of social sustainability in the supply chain: an example of footwear industry from an emerging economy. Sustain. Prod. Consum..

[57] Çankaya, Sibel Yildiz, Sezen Bulent (2019). Effects of green supply chain management practices on sustainability performance. J. Manuf. Technol. Manag..

[58] Mani V., Jabbour C.J.C., Mani K.T. (2020). Supply chain social sustainability in small and medium manufacturing enterprises and firms' performance: empirical evidence from an emerging Asian economy. Int. J. Prod. Econ..

[59] Govindan K., Rajeev A., Padhi S.S., Pati R.K. (2020). Supply chain sustainability and performance of firms: a meta-analysis of the literature. Transport. Res. E Logist. Transport. Rev..

[60] Karaosman Hakan, Perry Patsy, Brun Alessandro, Morales-Alonso Gustavo (2020). Behind the runway: extending sustainability in luxury fashion supply chains. J. Bus. Res..

[61] Agyabeng-Mensah Yaw, Ahenkorah Esther, Afum Ebenezer, Dacosta Essel, Tian Zhongxing (2020). Green warehousing, logistics optimization, social values and ethics and economic performance: the role of supply chain sustainability. Int. J. Logist. Manag..

[62] Bag Surajit, Telukdarie Arnesh, Pretorius JH Ch, Gupta Shivam (2021). Industry 4.0 and supply chain sustainability: framework and future research directions. Benchmark Int. J..

[63] Baghizadeh Komeyl, Pahl Julia, Hu Guiping (2021). Closed-loop supply chain design with sustainability aspects and network resilience under uncertainty: modelling and application. Math. Probl Eng..

[64] Bappy Mahathir Mohammad, Ali Syed Mithun, Kabir Golam, Paul Sanjoy Kumar (2019). Supply chain sustainability assessment with Dempster-Shafer evidence theory: implications in cleaner production. J. Clean. Prod..

[65] Bastas A., Liyanage K. (2019). Integrated quality and supply chain management business diagnostics for organizational sustainability improvement. Sustain. Prod. Consum..

[66] Chowdhury M.M.H., Quaddus M.A. (2021). Supply chain sustainability practices and governance for mitigating sustainability risk and improving market performance: a dynamic capability perspective. J. Clean. Prod..

[67] Hannibal Claire, Kauppi Katri (2019). Third party social sustainability assessment: is it a multi-tier supply chain solution?. Int. J. Prod. Econ..

[68] Karmaker Chitra Lekha, Ahmed Tazim, Ahmed Sayem, Ali Syed Mithun, Abdul Moktadir Md, Kabir Golam (2021). Improving supply chain sustainability in the context of COVID-19 pandemic in an emerging economy: exploring drivers using an integrated model. Sustain. Prod. Consum..

[69] Khan Syed Abdul Rehman, Karim Zkik, Belhadi Amine, Kamble Sachin S. (2021). Evaluating barriers and solutions for social sustainability adoption in multi-tier supply chains. Int. J. Prod. Res..

[70] León-Bravo V., Caniato F., Caridi M. (2019). Sustainability in multiple stages of the food supply chain in Italy: practices, performance and reputation. Operations Management Research.

[71] Orji I.J., Liu S. (2020). A dynamic perspective on the key drivers of innovation-led lean approaches to achieve sustainability in manufacturing supply chain. Int. J. Prod. Econ..

[72] Jabbour Charbel Jose Chiappetta, Jabbour Ana Beatriz Lopes de Sousa, Sarkis Joseph (2019). Unlocking effective multi-tier supply chain management for sustainability through quantitative modeling: lessons learned and discoveries to be made. Int. J. Prod. Econ..

[73] Krishnan, Ramesh, Agarwal Renu, Bajada Christopher, Arshinder K. (2020). "Redesigning a food supply chain for environmental sustainability–An analysis of resource use and recovery.". J. Clean. Prod..

[74] Cabernard Livia, Pfister Stephan, Hellweg Stefanie (2019). A new method for analyzing sustainability performance of global supply chains and its application to material resources. Sci. Total Environ..

[75] Gardner Toby A., Benzie Magnus, Börner Jan, Dawkins Elena, Fick Stephen, Garrett Rachael, Godar Javier (2019). Transparency and sustainability in global commodity supply chains. World Dev..

[76] Motlagh Hosseini, Mahdi Seyyed, Nouri-Harzvili Mina, Choi Tsan-Ming, Samira Ebrahimi (2019). Reverse supply chain systems optimization with dual channel and demand disruptions: sustainability, CSR investment and pricing coordination. Inf. Sci..

[77] Xu Ming, Cui Yuanyuan, Hu Meng, Xu Xinkai, Zhang Zhechi, Liang Sai, Qu Shen (2019). Supply chain sustainability risk and assessment. J. Clean. Prod..

[78] D'Eusanio Manuela, Zamagni Alessandra, Petti Luigia (2019). Social sustainability and supply chain management: methods and tools. J. Clean. Prod..

[79] Negri Marta, Cagno Enrico, Colicchia Claudia, Sarkis Joseph (2021). Integrating sustainability and resilience in the supply chain: a systematic literature review and a research agenda. Bus. Strat. Environ..

[80] Zavala-Alcívar Antonio, Verdecho María-José, Alfaro-Saiz Juan-José (2020). A conceptual framework to manage resilience and increase sustainability in the supply chain. Sustainability.

[81] Kalantary Majid, Saen Reza Farzipoor (2019). Assessing sustainability of supply chains: an inverse network dynamic DEA model. Comput. Ind. Eng..

[82] Neri Alessandra, Cagno Enrico, Lepri Marco, Trianni Andrea (2021). A triple bottom line balanced set of key performance indicators to measure the sustainability performance of industrial supply chains. Sustain. Prod. Consum..

[83] Gupta Himanshu, Kusi-Sarpong Simonov, Rezaei Jafar (2020). Barriers and overcoming strategies to supply chain sustainability innovation. Resour. Conserv. Recycl..

[84] Bai Qingguo, Xu Jianteng, Chauhan Satyaveer S. (2020). Effects of sustainability investment and risk aversion on a two-stage supply chain coordination under a carbon tax policy. Comput. Ind. Eng..

[85] Sharma M., Kamble S., Mani V., Sehrawat R., Belhadi A., Sharma V. (2021). Industry 4.0 adoption for sustainability in multi-tier manufacturing supply chain in emerging economies. J. Clean. Prod..

[86] Silva Minelle E., Marina D. Figueiredo (2020). Practicing sustainability for responsible business in supply chains. J. Clean. Prod..

[87] Jadhav A., Orr S., Malik M. (2019). The role of supply chain orientation in achieving supply chain sustainability. Int. J. Prod. Econ..

[88] Malak-Rawlikowska Agata, Majewski Edward, Wąs Adam, Borgen Svein Ole, Csillag Peter, Donati Michele, Freeman Richard (2019). Measuring the economic, environmental, and social sustainability of short food supply chains. Sustainability.

[89] Vittersø G., Torjusen H., Laitala K., Tocco B., Biasini B., Csillag P., Wavresky P. (2019). Short food supply chains and their contributions to sustainability: participants' views and perceptions from 12 European cases. Sustainability.

[90] Sharma Y.K., Mangla S.K., Patil P.P., Liu S. (2019). When challenges impede the process: for circular economy-driven sustainability practices in food supply chain. Manag. Decis..

[91] Munny Azmina Akter, Ali Syed Mithun, Kabir Golam, Abdul Moktadir Md, Rahman Towfique, Mahtab Zuhayer (2019). Enablers of social sustainability in the supply chain: an example of footwear industry from an emerging economy. Sustain. Prod. Consum..

[92] Mejías Ana M., Bellas Roberto, Pardo Juan E., Enrique Paz (2019). Traceability management systems and capacity building as new approaches for improving sustainability in the fashion multi-tier supply chain. Int. J. Prod. Econ..

[93] Sarkis Joseph (2020). Supply chain sustainability: learning from the COVID-19 pandemic. Int. J. Oper. Prod. Manag..

[94] Suhi Saima Ahmed, Enayet Rafid, Haque Tasmiah, Ali Syed Mithun, Abdul Moktadir Md, Paul Sanjoy Kumar (2019). Environmental sustainability assessment in supply chain: an emerging economy context. Environ. Impact Assess. Rev..

[95] Lai Xiaofan, Yi Tao, Wang Fan, Zou Zongbao (2019). Sustainability investment in maritime supply chain with risk behavior and information sharing. Int. J. Prod. Econ..

[96] Shi Xiutian, Chan Hau-Ling, Dong Ciwei (2018). Value of bargaining contract in a supply chain system with sustainability investment: an incentive analysis. IEEE Transactions on Systems, Man, and Cybernetics: Systems.

[97] Al-Okaily Manaf, Alkayed Hani, Aws Al-Okaily (2024). Does XBRL adoption increase financial information transparency in digital disclosure environment? Insights from emerging markets. International Journal of Information Management Data Insights.

[98] Elgedawy M.N. (2024). The relationship between data governance and organizational performance: the mediating effect of explainable artificial intelligence. Arab Journal of Administrative Sciences.

[99] Al-Okaily M. (2024). Advancements and forecasts of digital taxation information systems usage and its impact on tax compliance: does trust and awareness make difference?. J. Financ. Report. Account..

[100] Hasan F., Al-Okaily M., Choudhury T., Kayani U. (2023). A comparative analysis between FinTech and traditional stock markets: using Russia and Ukraine war data. Electron. Commer. Res..

[101] Al-Okaily M. (2024). So what about the post-COVID-19 era?: do users still adopt FinTech products?. Int. J. Hum. Comput. Interact..

[102] Al-Okaily M. (2024). Attitudes toward the adoption of accounting analytics technology in the digital transformation landscape. J. Account. Organ. Change.

[103] Al-Okaily M. (2024). Implications of the COVID-19 pandemic on continuance usage of electronic tax declaration platforms: extending classical UTAUT model. Digital Policy. Regulation and Governance.

[104] Al-Sartawi A., Sanad Z., Momany M.T., Al-Okaily M. (2023). European, Asian, Middle Eastern, North African Conference on Management & Information Systems.

[105] Qatawneh N., Al-Okaily A., Al-Okaily M., Ur Rehman S. (2024). "Exploring the antecedent factors of continuous intention to use mobile money: insights from emerging markets", Digital Policy. Regulation and Governance.

[106] El-Sayed A.H. (2023). The relationship between prior business ownership experience and opportunity identification: the role of cognitive styles. Arab Journal of Administrative Sciences.

[107] Younis H., Sundarakani B., O’Mahony B. (2020). Investigating the relationship between green supply chain management and corporate performance using a mixed method approach: Developing a roadmap for future research. IIMB Management Review.

[108] Al-Okaily M., Boshnak H., Alkayed H., Shehadeh E., Alqam M. (2024). From traditional to digital: the role of XBRL adoption in improving financial statements transparency. Global Knowledge, Memory and Communication.

[109] Younis H., Sundarakani B., Alsharairi M. (2022). Applications of artificial intelligence and machine learning within supply chains: systematic review and future research directions. J. Model. Manag..

[110] Alahmed M.A., Alasfour M.G., Salifu E. (2023). Users' informativity and effective communication: the banking field in Kuwait. Arab Journal of Administrative Sciences.

[111] Yousefi S., Tosarkani B.M. (2022). An analytical approach for evaluating the impact of blockchain technology on sustainable supply chain performance. Int. J. Prod. Econ..

[112] Alsmadi A.A., Al-Okaily M., Alrawashdeh N., Al-Gasaymeh A., Moh’d Al-hazimeh A., Zakari A. (2023). A bibliometric analysis of green bonds and sustainable green energy: evidence from the last fifteen years (2007–2022). Sustainability.

